# Bioactive Properties of Venoms Isolated from Whiptail Stingrays and the Search for Molecular Mechanisms and Targets

**DOI:** 10.3390/ph17040488

**Published:** 2024-04-11

**Authors:** Craig A. Doupnik, Carl A. Luer, Catherine J. Walsh, Jessica Restivo, Jacqueline Xinlan Brick

**Affiliations:** 1 Department of Molecular Pharmacology & Physiology, Morsani College of Medicine, University of South Florida, Tampa, FL 33612, USA; 2 Marine Biomedical Research Program, Mote Marine Laboratory, Sarasota, FL 34236, USA; caluer@mote.org; 3 Marine Immunology Program, Mote Marine Laboratory, Sarasota, FL 34236, USA; cjwalsh@mote.org (C.J.W.); jrestivo@wustl.edu (J.R.); 4 Department of Biology, College of Arts & Sciences, Oberlin College and Conservatory, Oberlin, OH 44074, USA; jackiebrick222@gmail.com

**Keywords:** apoptosis, cell adhesion, cell growth, fibrosarcoma, galectin-like proteins, necrosis, stingray, toxin, venom

## Abstract

The venom-containing barb attached to their ‘whip-like’ tail provides stingrays a defensive mechanism for evading predators such as sharks. From human encounters, dermal stingray envenomation is characterized by intense pain often followed by tissue necrosis occurring over several days to weeks. The bioactive components in stingray venoms (SRVs) and their molecular targets and mechanisms that mediate these complex responses are not well understood. Given the utility of venom-derived proteins from other venomous species for biomedical and pharmaceutical applications, we set out to characterize the bioactivity of SRV extracts from three local species that belong to the *Dasyatoidea* ‘whiptail’ superfamily. Multiple cell-based assays were used to quantify and compare the in vitro effects of these SRVs on different cell lines. All three SRVs demonstrated concentration-dependent growth-inhibitory effects on three different human cell lines tested. In contrast, a mouse fibrosarcoma cell line was markedly resistant to all three SRVs, indicating the molecular target(s) for mediating the SRV effects are not expressed on these cells. The multifunctional SRV responses were characterized by an acute disruption of cell adhesion leading to apoptosis. These findings aim to guide future investigations of individual SRV proteins and their molecular targets for potential use in biomedical applications.

## 1. Introduction

Venoms are complex mixtures of bioactive secretions that have evolved independently numerous times throughout the animal kingdom in both invertebrate and vertebrate phyla [1,2]. Each venom is composed of numerous compounds that possess a variety of bioactivities (i.e., proteolytic, hemotoxic, neurotoxic, or cytotoxic activity), and collectively serve a variety of biological functions for the host animal, including predation, defense from predators, and competitor deterrence [1,2]. Although several individual venom compounds have been developed into therapeutic drugs, including blood pressure regulators, blood thinners (anticoagulants), and pain relievers associated with specific ion channel blockers, none to date have originated from stingray venom (SRV) [3,4,5,6].

Unlike most venomous animals whose venom glands are distinct well-defined organs, SRV secretion emanates from diffuse masses of cells that lie between the epidermis and dermis along either side of the midventral ridge of a serrated cartilaginous barb [7,8,9,10,11]. Stingray venom glands are holocrine glands, where venom secretion results from physical rupture and disintegration of the venom-containing gland cells when the integumentary sheath is shed from the barb during the mechanical whip-like action of the tail [12,13]. Obtaining the diffuse crude venom secretions from stingray barbs is challenging, and this provides a likely reason why SRVs have been less studied compared to those from other venomous species.

Bioassays for screening and characterizing venom activity typically measure changes in cell viability (toxicity), migration, and growth using biochemical markers with colorimetric assays and impedance-based biosensing techniques [14,15]. Applying these methods to cultured cells derived from tissues to which venom compounds are typically exposed (such as skin, nerve, muscle, and blood) provides an initial assessment and characterization of SRV bioactivities. Proteomic and transcriptomic approaches are now extending these approaches by identifying the complement of expressed SRV proteins and peptides attributable to known venom functions that then holistically contribute to the pathophysiological responses elicited by crude venom extracts [16,17,18,19].

Our study reported here characterizes the bioactive properties of SRV collected from three local species of stingrays commonly found in nearshore waters off the southwest coast of Florida: the Atlantic stingray (*Hypanus sabinus*, *Dasyatidae* family), the spotted eagle ray (*Aetobatus narinari*, *Aetobatidae* family), and the cownose ray (*Rhinoptera bonasus*, *Rhinopteridae* family). The selected target cells for characterizing the SRV effects represent cell types derived from tissues to which these SRVs might normally respond (i.e., of skin, blood, and neural origin); specifically, they include a human dermal fibroblast cell line (HDFa), a human neuroblastoma cell line (SH-SY5Y), a human T-cell leukemia cell line (Jurkat E6-1), and a murine fibrosarcoma cell line (WEHI-164) (cf. Table 1).

## 2. Results

### 2.1. Biochemical Characterization of Isolated SRV Proteins

**Figure 1:**
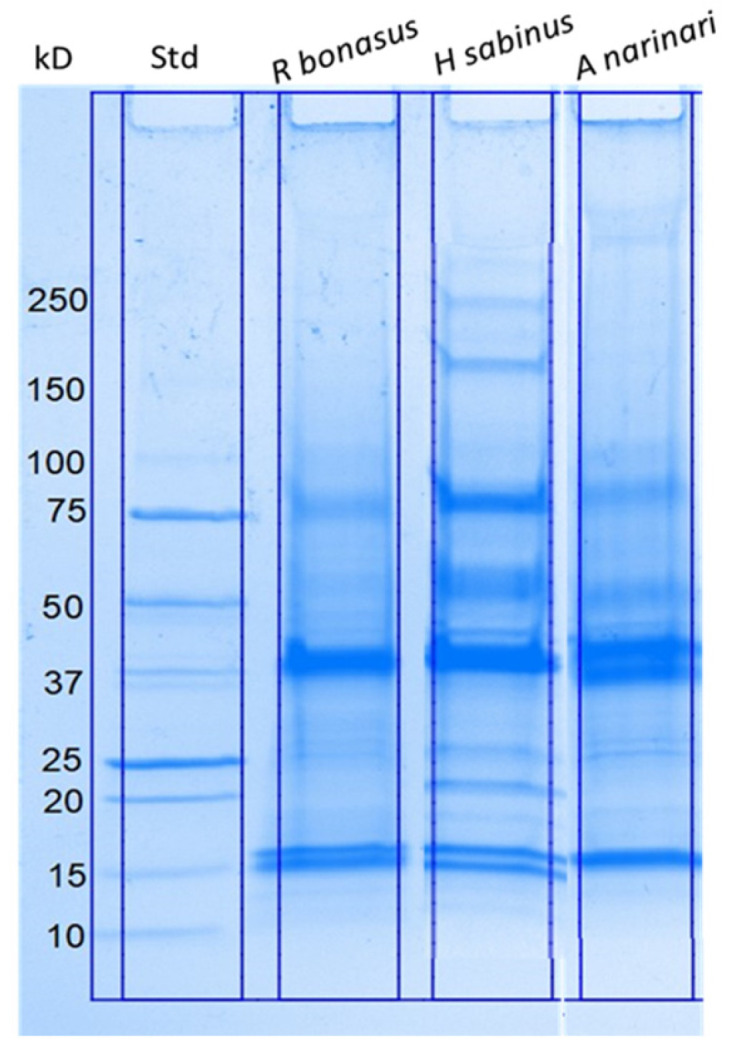
SDS–polyacrylamide gel electrophoretic separation of crude SRV proteins obtained from three distinct stingray species. Lane 1: Molecular weight standards. Lane 2: Cownose ray (*R. bonasus*). Lane 3: Atlantic stingray (*H. sabinus*). Lane 4: Spotted eagle ray (*A. narinari*). The amount of protein loaded per lane was 25 μg for each SRV.

Preparation of enriched SRV extracts from collected stingray spines was standardized to enable the assessment of possible species differences. Crude SRV protein samples from the Atlantic stingray, spotted eagle ray, and cownose ray were initially evaluated and compared by SDS-PAGE analysis (Figure 1). The resolved SRV proteins from each species ranged in molecular size from ~15 kD to greater than 250 kD, where the banding patterns were similar but species-specific. Major protein bands corresponding to approximately 16 kD, 38 kD, and 75 kD were present in all three venoms.

### 2.2. Bioactive Properties of SRVs on Cultured HDFa and SH-SY5Y Cells

**Figure 2:**
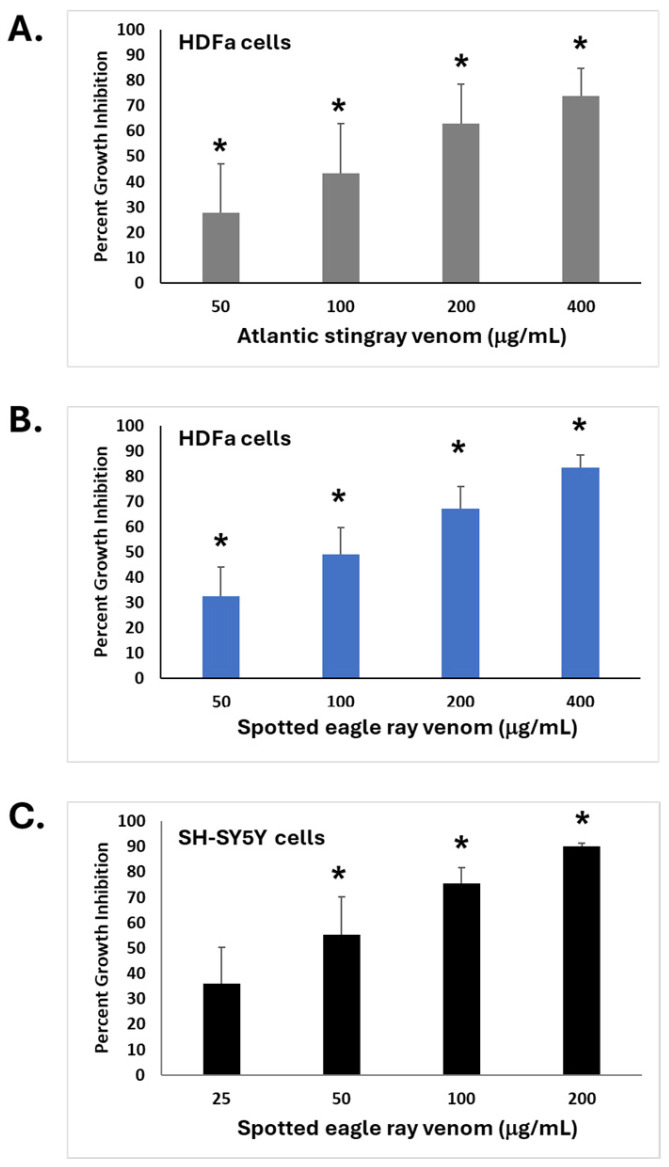
Inhibition of cell growth caused by exposure to SRVs. (**A**) HDFa cells treated with Atlantic stingray venom (mean ± S.E.M., *n* = 3) at protein concentrations of 50, 100, 200, and 400 µg/mL. (**B**) HDFa cells treated with spotted eagle ray venom (mean ± S.E.M, *n* = 4) at concentrations of 50, 100, 200, and 400 µg/mL. (**C**) SH-SY5Y cells treated with spotted eagle ray venom (mean ± S.E.M., *n* = 6) at concentrations of 25, 50, 100, and 200 µg/mL. The percent growth inhibition compared to untreated control cells (0 μg/mL) was determined using the MTT assay following a 72 h treatment period for each condition. * Significantly different from untreated control cells as determined by Student’s *t*-test (*p* < 0.05).

To screen the bioactivity of SRV extracts on cultured HDFa and SH-SY5Y cells in vitro, cell cultures were treated with a range of SRV protein concentrations for a 72 h exposure period and then assayed for cell growth using the MTT assay [15]. The concentration-dependent effects of Atlantic stingray venom and spotted eagle ray venom are shown in Figure 2 and presented as ‘percent growth inhibition’ relative to untreated control cells. Both SRVs inhibited cell growth when compared to untreated control groups and were dependent on the concentration of the SRV tested.

For HDFa cells exposed to Atlantic stingray venom (Figure 2A), all concentrations caused a significant reduction in cell growth compared to the untreated control cells (*p* < 0.05). The calculated concentration producing 50% inhibition (IC50) [20] was 98.4 μg/mL. For HDFa cells treated with spotted eagle ray venom (Figure 2B), all concentrations similarly caused a significant reduction in cell growth compared to untreated control cells (*p* < 0.05). The calculated IC50 for spotted eagle ray venom-mediated cell growth inhibition of HDFa cells was 116.4 μg/mL.

For the neuronally derived SH-SY5Y cells (Figure 2C), the growth inhibitions caused by 50, 100, and 200 µg/mL spotted eagle ray venom were each significantly greater than in the untreated control (*p* < 0.05), where 25 µg/mL treatment did not cause a significant difference from untreated control cells. The calculated IC50 for spotted eagle ray venom-mediated cell growth inhibition of SH-SY5Y cells was 23.7 μg/mL. Thus, both cell lines exhibited sensitivity to SRV-mediated growth inhibition as determined by the MTT assay; the IC50 was lower when SH-SY5Y cells were used as the target.

### 2.3. Concentration-Dependent Effects of SRV on Cell Morphology

**Figure 3:**
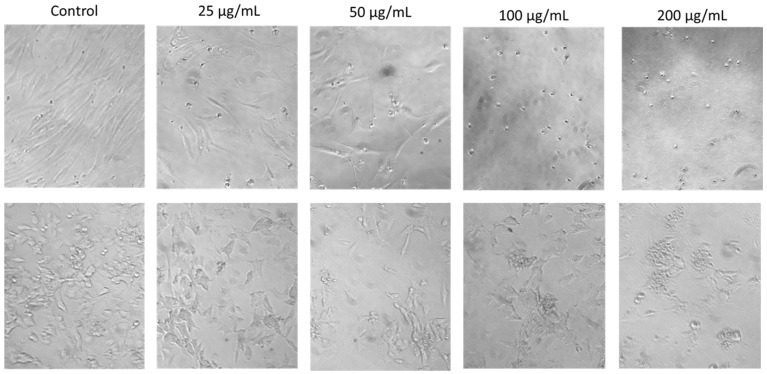
Morphological effects of eagle ray venom on cultured HDFa cells (**top row**) compared to cultured SH-SY5Y cells (**bottom row**). The two cell types were each treated with a range of SRV concentrations for a 72 h exposure period. Magnification, 10×.

The effects of SRV treatment on cell morphological features could also be readily observed under the microscope and were evident at the lowest SRV concentrations tested after the 72 h exposure period (Figure 3). For both HDFa and SH-SY5Y cells, a decrease in cell density and an increase in small, round cells were readily visualized with increasing spotted eagle ray venom concentrations. At the two higher concentrations (100 and 200 µg/mL), very few intact cells were observed, and cells mainly consisted of small, rounded cells and cellular debris. These morphological features are consistent with cells undergoing apoptosis.

### 2.4. Time Course for SRV-Mediated Effects on HDFa Cell Cultures

**Figure 4:**
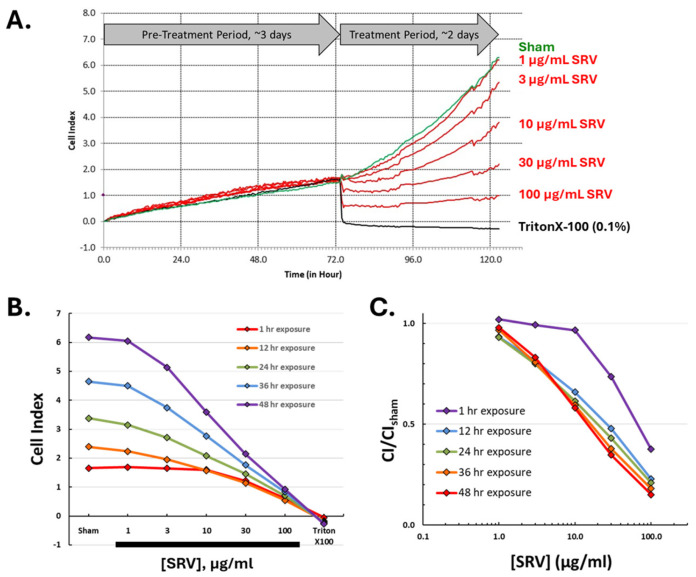
Time course for Atlantic stingray venom effects on HDFa cells maintained in culture. (**A**) The Cell Index (normalized impedance) was recorded in 96-well E-plates every 15 min and plotted over a 5-day time period. The slow, near-linear increase in the Cell Index that occurred during the 3-day pre-treatment period reflects the proliferation and adherence of HDFa cells that result in an increase in impedance. The Cell Index values at time ‘zero’ were normalized for time course comparisons caused by the different experimental groups over the 2-day treatment period: sham (green trace), SRV treatments (red traces), and TritonX-100 (black trace). (**B**) The Cell Index values measured at increasing durations of SRV exposure (1, 12, 24, 36, and 48 h) are plotted for each concentration of SRV tested. (**C**) The derived SRV concentration–response effects are shown after acute SRV exposure (1 h) and increasing SRV exposure durations (12, 24, 36, and 48 h). The Cell Index values from each SRV treatment group were normalized to the sham control value for comparisons of the concentration-dependent responses at different exposure periods.

To better resolve the time course for SRV-mediated effects on cell growth, a biosensor-based cell impedance analysis was performed on cultured HDFa cells treated with Atlantic stingray venom at varying concentrations. After initial cell seeding in the biosensor culture wells, cell growth was recorded every 15 min over a 3-day pre-treatment period. Shown in Figure 4, the near-linear increase in impedance during the pre-treatment period reflects the time course for cell proliferation and an increase in adherent HDFa cells (i.e., rate of cell growth). On day 4, cells were exposed to varying concentrations of SRV and the impedance was recorded for a 48 h treatment period. Under the culturing conditions used, HDFa cells entered near log phase growth around days 3–4 when the SRV treatment was initiated (Figure 4).

Time course analysis of the 48 h SRV exposure period revealed two distinct effects caused by SRV treatment that were dependent on the concentration and exposure time. At the higher SRV concentrations tested (30–100 μg/mL), there was a distinct acute effect that was characterized by a very rapid decrease in impedance (starting immediately) that was complete within 1 h of treatment and reflects an abrupt decrease in cell adherence (i.e., cell detachment). The rapid decrease caused by high SRV concentrations resembled the time course caused by Triton-X treatment that was used as a positive control for acute full cell detachment. The impedance IC50 for the rapid acute effect, measured after 1 h of exposure for all concentrations, was 74.2 μg/mL (Figure 4C).

At the lower SRV concentrations that did not produce rapid cell detachment (i.e., 3–10 μg/mL), there was still a notably slower rate of cell growth over the full 48 h exposure period. This finding is consistent with the inhibition of cell growth observed in the MTT assay following 72 h SRV exposures (cf. Figure 2). The impedance IC50 after 48 h of exposure was 16.7 μg/mL (Figure 4C).

**Figure 5:**
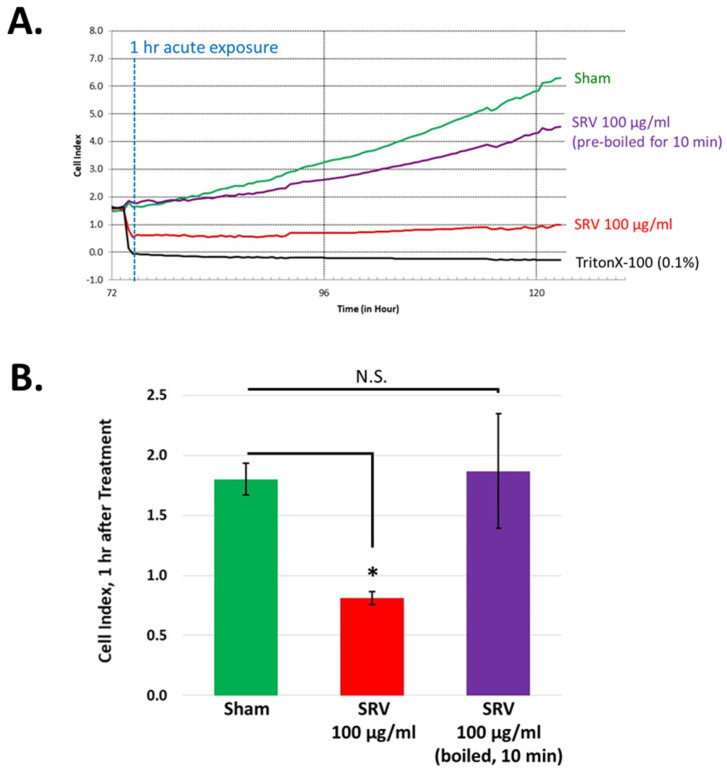
Heat denaturation of SRV abolishes the rapid cellular detachment effect. (**A**) HDFa cells in log phase growth were either untreated (sham, green trace), treated with 100 μg/mL Atlantic stingray venom (red trace), or treated with 100 μg/mL Atlantic stingray venom subjected to high heat (purple trace). TritonX-100 treatment (black trace) served as a positive control for cell detachment. (**B**) HDFa cell adherence measured 1 h after the start of the treatment periods is shown for each experimental group (mean ± S.D., *n* = 3, * *p* < 0.05, N.S. denotes not statistically significant).

The bioactivity of SRVs is known to be thermolabile, where applying hot water to the site of injury is recommended to mitigate the acute pain and tissue necrosis associated with stingray envenomation [21,22,23]. To test the thermolability of SRV in our experiments, Atlantic stingray venom was subjected to a high temperature (100 °C) for 10 min to denature the venom protein components. As shown in Figure 5, heat treatment abolished the rapid cell detachment effect caused by 100 μg/mL SRV. In contrast, the growth-inhibitory effect of the SRV was still present after heat treatment (Figure 5B). These findings indicate that protein component(s) in Atlantic stingray venom mediate the rapid cell detachment effect (heat-sensitive), whereas other venom components insensitive to heat can contribute towards the growth-inhibitory effect.

### 2.5. Bioactive Effects of SRVs on Additional Tumor Cell Types

**Figure 6:**
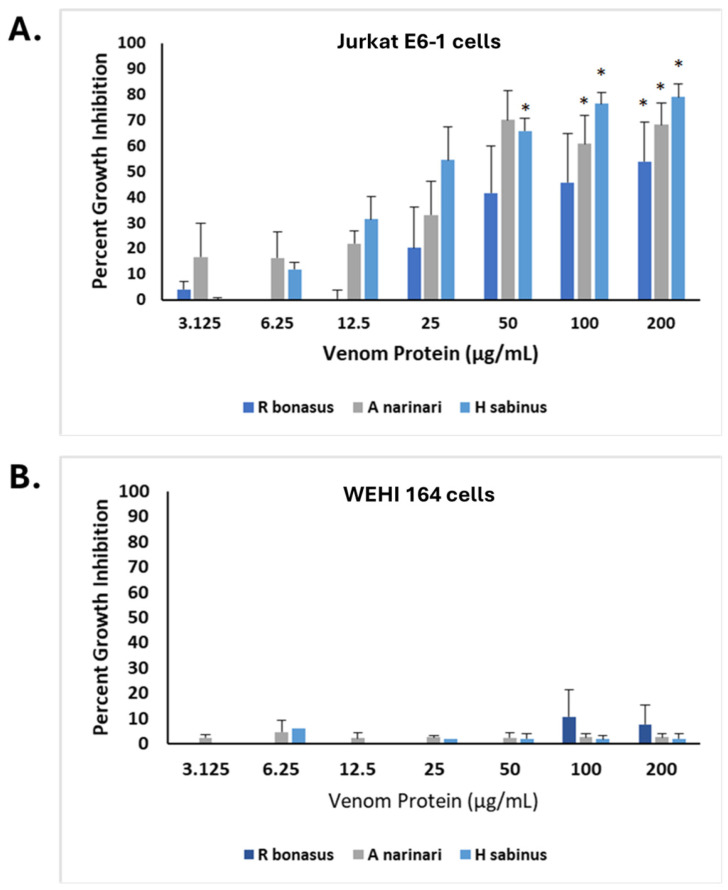
SRV-mediated inhibition of Jurkat E6-1 cell growth and insensitivity of WEHI 164 cells. (**A**) SRVs from cownose rays (*R. bonasus*), spotted eagle rays (*A. narinari*), and Atlantic stingrays (*H. sabinus*) were used to treat Jurkat E6-1 cells for a 24 h exposure period at varying concentrations. The percent growth inhibition compared to untreated control cells (0 μg/mL) was determined using the MTT assay (mean ± S.E.M.; *R. bonasus* venom, *n* = 6; *A. narinari* venom; *n* = 8, *H. sabinus* venom, *n* = 4). (**B**) WEHI 164 fibrosarcoma cells were treated with SRVs under the same conditions described for Jurkat E6-1 cells (mean ± S.E.M., *n* = 3 for each SRV). * Significantly different from untreated control cells as determined by Student’s *t*-test (*p* < 0.05).

Our initial experiments demonstrated the effects of SRV on two cell types: HDFa and SH-SY5Y. To explore the effects of SRV on different tumor cell types, a non-adherent human T-lymphocyte tumor cell line (Jurkat E6-1 cells) was examined in addition to a chemically induced mouse fibrosarcoma cell line (WEHI-164 cells). Using the MTT cell growth assay, the comparative effects of spotted eagle ray, Atlantic stingray, and cownose ray venoms are shown in Figure 6. All three SRVs demonstrated significant growth-inhibitory effects on Jurkat E6-1 cells (Figure 6A), and generally had the following rank order of potency: spotted eagle ray > Atlantic stingray > cownose ray. Venom from the Atlantic stingray at each of the 50 µg/mL, 100 µg/mL, and 200 µg/mL treatment concentrations was significantly different from the untreated control (*p* < 0.05). With eagle ray venom, 100 µg/mL and 200 µg/mL treatments were significantly different from the untreated control (*p* < 0.05). Cownose ray venom caused a significant difference at 200 µg/mL (*p* < 0.05) compared to the untreated control. The SRV concentration dependencies approximated the growth inhibition observed for both HDFa and SH-SY5Y cells (cf. Figure 2).

Interestingly, however, the mouse WEHI 164 fibrosarcoma cell line was found to be insensitive to all three SRVs tested following the 24 h exposure period (Figure 6B). Using the same SRV concentrations tested in the other cell lines, no venom treatments resulted in significantly different WEHI 164 cell growth when compared to untreated controls (Figure 6B).

### 2.6. SRVs Cause Apoptosis of Jurkat E6-1 Cells

**Figure 7:**
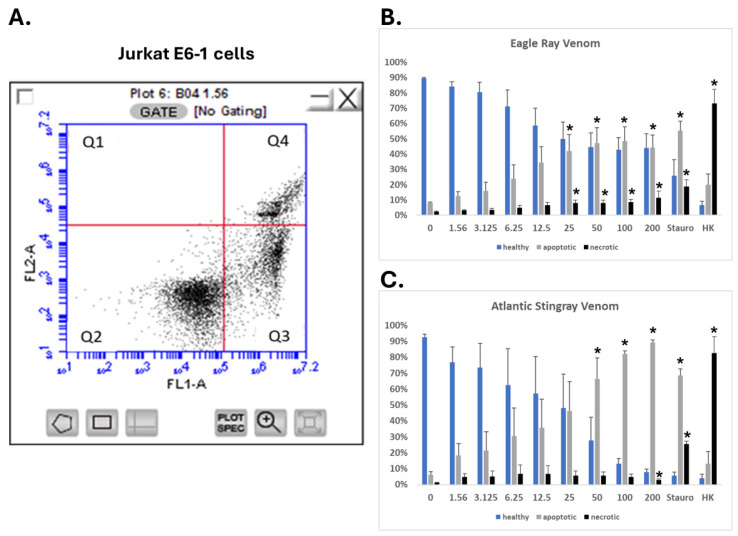
SRV-mediated apoptosis and necrosis of Jurkat E6-1 cells. (**A**) Representative flow cytometry analysis of Jurkat E6-1 cells assessed with FITC-labeled annexin V binding (FL1-A channel) and propidium iodide staining (FL2-A channel). Healthy viable cells cluster in Quadrant 2 (Q2), apoptotic cells cluster in Quadrant 3 (Q3), and dead or necrotic cells cluster in Quadrant 4 (Q4). (**B**,**C**) The percentages of Jurkat E6-1 cells (mean ± S.E.M.) characterized as ‘healthy’, ‘apoptotic’, or ‘necrotic’ following a 24 h exposure to eagle ray venom (b) or Atlantic stingray venom (c) are plotted over the range of concentrations tested (0–200 μg/mL). Staurosporine treatment (Stauro) was used as a positive control for apoptosis and heat-killing (HK) of cells was used as a positive control for cell necrosis (95 °C for 30 min). * Significantly different than untreated control as determined using Student’s *t*-test (*p* < 0.05).

To further explore the growth-inhibitory effects of SRVs on Jurkat E6-1 cells, these non-adherent cells were assayed for apoptosis and necrosis using flow cytometry [24]. Jurkat E6-1 cells were exposed for 24 h to either spotted eagle ray venom or Atlantic stingray venom at varying concentrations, then assayed for apoptosis and cell death. The results shown in Figure 7 illustrate an SRV concentration-dependent decrease in healthy cells with a concomitant increase in both apoptosis and necrosis for both SRV treatments. The threshold concentration producing apoptosis was 25 μg/mL for spotted eagle ray venom versus 50 μg/mL for Atlantic stingray venom, with the onset of necrosis also occurring at a significantly lower concentration with spotted eagle ray venom treatment.

## 3. Discussion

### 3.1. The Evolution of SRVs as a Well-Conserved Defense Response

The evolutionary emergence of stingray spines dates back to the Late Cretaceous, with expansion occurring in the Late epoch, 100.5 to 66 million years ago [25]. The temporal correlation with the expansion of large sharks during this time period suggests that predatory pressures on early rays led to the adaptive development and resulting venom-laced barb that is characteristic of today’s Myliobatoidei suborder [26]. The venomous spine appendage is found worldwide on ~220 stingray species inhabiting both fresh- and saltwater habitats. With the emergence of SRV as a noxious mixture of compounds to repel predators, the conserved and non-conserved components that have evolved across different stingray species are currently not well understood.

The noxious and mostly non-lethal experience of stingray envenomation is recognized from human encounters and assumed to be a similar experience for an attacking stingray predator. The defensive tail-whip reflex of the stingray causes two distinct events to their targets: (1) dermal laceration caused by the serrated barb that provides deep tissue access locally and to the microcirculation, and (2) envenomation of SRV components into the penetrated tissue via holocrine release from venom glands located on the barb [8,10]. Envenomation is typically localized but can be systemic depending on the anatomical location of the mechanical impalement and extent of venom deposition. Lethal outcomes are rare in humans and generally occur with systemic envenomation impacting cardiovascular system function [27].

### 3.2. The Bioactivity of Isolated SRVs Is Well Conserved across Diverse Species

We examined the properties of SRV extracts isolated from three species belonging to three distinct stingray families (*Dasyatidae, Aetobatidae,* and *Rhinopteridae*) [28]. The bioactivity of their SRV highlights a generally conserved activity across this large and diverse family of whiptail stingrays. Our standardized procedures for collecting, processing, and assaying SRV bioactivity on mammalian cells in culture revealed consistent and reproducible responses produced by each venom. Based on our findings, the sequelae of events associated with SRV exposure initiate immediately with cellular events that disrupt cell adhesion, leading to cell detachment, followed by growth inhibition, apoptosis, and necrosis.

The biochemical profiles from SDS-PAGE separation of the crude SRV extracts indicate the presence of both conserved proteins and proteins that are species-specific. This initial biochemical characterization of the collected SRV extracts did not include other analytical methods (i.e., HPLC or mass spectrometry) to resolve lower-abundance venom proteins, smaller venom peptides, and chemicals expected to be present in our samples. Furthermore, the SRV extracts were derived from ventral spine tissue that includes both the venom-producing secretory cells as well as other neighboring connective tissue that is unavoidably included with the collection method. We therefore cannot associate the origin of the biochemically identified proteins exclusively with venom proteins produced by the venom gland cells. Nevertheless, the SRV protein samples we examined are clearly enriched in venom proteins based on the collection method used and their demonstrated bioactivities.

### 3.3. SRV Proteins Identified by Recent Venomic Studies

SRV has historically been viewed as a ‘neglected venom’ given the difficulty in obtaining significant amounts of venom for investigation. With the advent and application of multi-omic technologies, resolving the constellation of proteins in SRV tissue extracts has become increasingly feasible. Using proteomic and transcriptomic analysis of crude venom isolated from the marine, blue-spotted stingray *Neotrygon kuhlii*, 18 distinctive proteins were identified [16]. Two cystatin proteins (cystatin-1 and cystatin-2) represented the most abundant protein class (19% combined), followed by a galectin-like protein (12%), and then a peroxiredoxin-6 protein (10%). The other 14 proteins comprised the 59% remaining venom protein content. Cystatin proteins are known peptidase inhibitors found in many other venoms and thought to serve protective functions for other venom peptides and proteins. Interestingly, however, cystatin from snake venom has been shown to also inhibit cell growth and migration in mouse melanoma and human hepatocellular carcinoma cell lines in vitro [29]. Galectin proteins are a multifunctional class of proteins and are discussed in greater length below. Peroxiredoxin-6 is a ubiquitous bifunctional enzyme with both glutathione peroxidase and phospholipase A2 (PLA2) activities. PLA2 activity is of interest given the well-established toxic effects of PLA2 activity in many other well-characterized venoms.

Venomic approaches have also been applied to SRVs isolated from freshwater stingrays inhabiting South American rivers, which reportedly are more toxic than saltwater stingrays [30]. These studies reveal a plethora of venom protein components that also included cystatins and peroxiredoxin-6, where hyaluronidases were found in greatest abundance [17,18]. Hyaluronidases are a highly conserved component present in many venoms that promote tissue deposition and the spread of other venom components through the degradation of hyaluronic acid, a major glycosaminoglycan component of the extracellular matrix [1,2]. A total of 111 and 115 venom transcripts were identified from two separate freshwater species, *Potamotrygon amandae* and *Potamotrygon falkneri* (*Potamotrygonidae* family), respectively.

Finally, a recent tour de force study using integrative venomics of SRVs from three marine and two freshwater stingrays further highlights the complexity of SRV components [19]. Seventy shared venom transcripts belonging to known toxin families were linked to known functional bioactivity clusters that include pain, cardiotoxicity, and hemorrhage. Venom transcripts for bibrotoxin- and cholecystotoxin-like proteins were identified as candidate pain-inducing proteins in SRV, acting via G-protein coupled receptor (GPCR) neuronal signaling pathways. The large number of identified SRV components with known toxin functions points towards a holistic envenomation model where clustered components work synergistically to produce the pathophysiological responses that characterize SRV injury (i.e., pain, inflammation, and tissue necrosis).

### 3.4. Mining SRV Proteins for Molecular Targets and Biomedical Applications

With the large number of SRV components identified by the venomic investigations, ‘next steps’ include studies investigating the bioactive contributions of individually identified SRV components to validate or invalidate their role and magnitude in the stingray envenomation responses causing acute pain, altered hemostasis, and tissue necrosis. In the context of the reported SRV venomic findings, our results support multiple components contributing to the observed in vitro bioactivities that include identified known toxins targeting extracellular matrix proteins (e.g., hyaluronidases and metalloproteinases) and cell membrane proteins (e.g., three-finger toxins and K^+^ channel toxins). Identified SRV proteins that are not classified and annotated as ‘toxins’ may also contribute to the biological response. We provide an example of this below for the abundant SRV galectin-like proteins that are not currently classified as toxins. This provides just one example of future directions aimed to functionally assess the contribution of individual SRV proteins (and their protein families) in the envenomation responses.

### 3.5. An SRV Galectin Toxin Hypothesis

The proteomic identification of a galectin-like protein as the second-most abundant protein in *N. kuhlii* venom, and also reportedly present in teleost venom [11,16], is especially intriguing given the well-established multifunctional characteristics of vertebrate galectins [31,32]. Several galectins have also been identified in the venom of other marine stingrays, but currently are not classified as toxins or members of a venom toxin family [19]. When considered together as a galectin protein family, the galectins identified in the venom of the marine ray *Dasyatis pastinaca* were the most abundant protein family represented in the venomic results [19].

Galectins are members of the larger lectin family of carbohydrate-binding proteins, where calcium-dependent (C-type) lectins are established toxins and are classified as members of a venom lectin family of proteins [33]. In contrast, galectins are calcium-independent and are currently not classified as toxins, yet are known to mediate many of the biological effects observed in our SRV experiments and reported in the literature. Given their reported abundance in marine SRV, galectins appear poised to be an important SRV component contributing to the biological responses associated with envenomation [31,32].

We propose the following hypothesis to serve as a mechanistic and testable model for future investigation. This model emanates from our experimental observations of three different SRVs and their bioactivity profiles aligning with known galectin functions, together with the unexpected insensitivity of WEHI-164 cells to all three SRVs tested.

***The Galectin Toxin Hypothesis***

**Figure 8:**
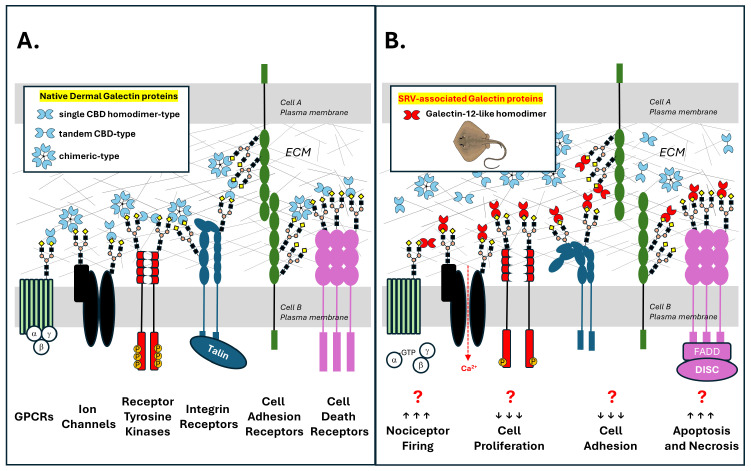
Conceptual diagram of SRV galectins targeting the native galectin lattice and affecting multiple cell membrane proteins via binding to their glycosylated and exposed β-galactoside structures. (**A**) In native tissues, a multivalent galectin lattice promotes clustering and physical coupling of glycosylated cell membrane proteins to the extracellular matrix (ECM) and neighboring transmembrane proteins. Normal homeostatic signaling is produced in part via these interactions. (**B**) Introduction of SRV galectin proteins during envenomation displaces and disrupts the native galectin lattice, resulting in altered cell signaling events mediated by the effected glycosylated transmembrane signaling protein targets.

1. Galectin-like proteins expressed in venom gland cells of marine stingrays are released locally into target tissues during barb-mediated envenomation.
2. SRV galectin-like proteins, via their carbohydrate-binding domains (CBDs), recognize and bind to β-galactoside glycan structures present on multiple N-linked and O-linked glycosylated membrane protein targets that are present on resident cells within the affected tissue (e.g., skin fibroblasts, neurons, and blood cells).
3. Binding of dimerized SRV galectin-like proteins to glycosylated target cell proteins promotes aberrant signaling by
  - severely disrupting the native galectin lattice that exists on the target cell glycoproteins [34];
  - promoting novel crosslinking and clustering of glycosylated transmembrane receptors and ion channels to initiate toxin-mediated downstream signaling events [35].
4. Collectively, these SRV galectin-mediated events either cause or contribute to the hallmark features of localized stingray injury, i.e., severe pain and dermal tissue necrosis (Figure 8).
5. We further hypothesize that the SRV-resistant characteristics of WEHI-164 cells are due to a distinct glycocalyx where either SRV galectin-like proteins do not recognize glycan targets on WEHI-164 cells, and/or the recognized glycan targets on HDFa, SH-SY5Y, and Jurkat-E6-1 cells are not expressed and presented by WEHI-164 cells. Abnormal glycosylation patterns are a common feature of malignant cells.

### 3.6. SRV Galectins

**Figure 9:**
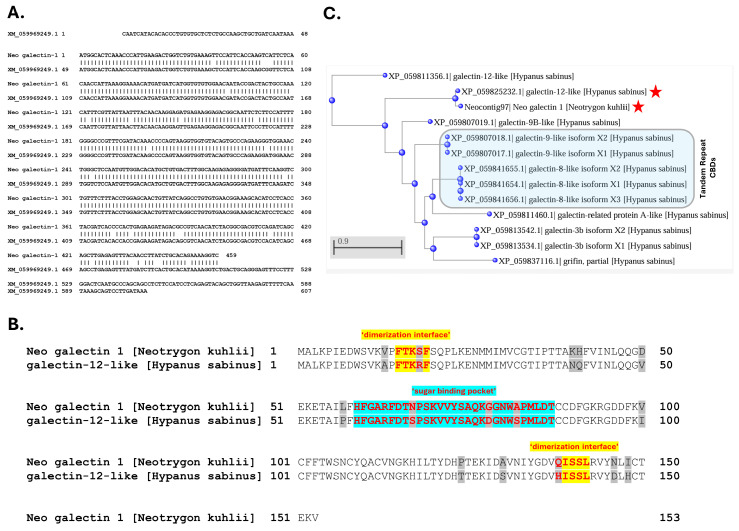
A venom-associated galectin-like protein homolog in the Atlantic stingray. (**A**) Nucleotide sequence alignment of Neocontig97 from *Neotrygon kuhlii* and the *Hypanus sabinus* mRNA RefSeq transcript (XM_059969249.1) identified by a BLAST database search. (**B**) Amino acid sequence alignment of the *Neotrygon kuhlii* galectin-1 protein and predicted *Hypanus sabinus* galectin-12-like protein. The conserved domains representing the dimerization interface sequences (yellow) and ‘sugar binding pocket’ (blue) (i.e., CBD) are highlighted with red font, and non-identical amino acids are highlighted in grey. Both proteins were classified as GLECT domain-containing proteins using the CD-Search interface of the NCBI conserved domain database, https://www.ncbi.nlm.nih.gov/cdd (accessed on 15 February 2024). The two proteins share 90% sequence identity. (**C**) Phylogenetic representation of predicted galectin proteins encoded by galectin-like genes annotated in the sHypSap1.hap1 genome assembly. The phylogenetic tree was constructed from a multiple sequence alignment of the proteins shown using COBALT and Phylogenetic Tree View with the following parameters: Fast Minimum Evolution, Maximum Sequence Distance = 0.90, and the Grishin protein algorithm. The results were then sorted by distance in ascending order. The venom-associated galectins are indicated by the two red stars.

The galectin-like transcript in *N. kuhlii* venom tissue (i.e., Neo galectin-1) and its encoded protein (the second-most abundant venom protein characterized) were originally identified by high sequence alignment with a galectin-like protein previously identified in the Japanese puffer fish (*Takifugu rubripes*) genome (UniprotKB ID: H2UTD9) [16,36]. A current BLAST search using the Neo galectin-1 nucleotide sequence identified a predicted transcript (XM_059969249.1) in the *H. sabinus* genome that encodes a 150 amino acid ‘galectin-12-like’ protein homolog (XP_059825232.1) (Figure 9). The Neo galectin-1 protein and *H. sabinus* galectin-12-like protein are both protomer-type galectins (i.e., contain a single CBD) and share 90% amino acid sequence identity (Figure 9B). Given the *N. kuhlii* proteomic results confirmed the presence of the Neo galectin-1 protein in *N. kuhlii* venom, we posit that the *H. sabinus* galectin-12-like protein homolog is similarly present in the venom of the Atlantic stingray.

The *H. sabinus* galectin-12-like protein is encoded by five exons located on chromosome 5 (LOC132393850) based on the sHypSap1.hap1 genome assembly (GCF_030144855.1). The assembled Atlantic stingray genome revealed a total of at least eight distinct galectin-like genes encoding 12 putative galectin-like protein isoforms (Figure 9C). Several of the *H. sabinus* galectin-like proteins encode protomer-type galectins (single CBD) with dimerization sequences at both N- and C-termini for homo-dimer formation. Both the *H. sabinus* galectin-8-like and galectin-9-like proteins are tandem repeat-type galectins, with each encoding two distinct CBDs separated by an amino acid linker sequence [31,32]. The venom-associated galectins may represent a distinct subfamily of galectin-like proteins.

### 3.7. Galectin Inhibitors in Cancer Therapeutics

Galectins have received considerable attention in cancer research given known differences in glycosylation patterns between normal and malignant tumor cells [37,38]. Cell surface glycan differences have been attributed to changes in cell adhesion and growth that represent hallmark features in cancer biology. Fourteen known galectin genes in humans participate in a broad range of galectin-specific regulatory processes that are altered in several diseases [32]. Their utility in cancer diagnosis and prognosis has also gained considerable interest [37]. Moreover, galectin selective inhibitors have recently received FDA approval for the treatment of conditions where elevated galectin levels are linked to clinical disease, and targeting them with inhibitors has shown therapeutic potential [39].

### 3.8. Venoms as a Source of Potential Anticancer Agents

The antiproliferative properties of venoms extracted from vertebrates and invertebrate species have been widely reported, and the potential application of venoms as anticancer agents has received considerable attention during the past decade, especially with snake venoms (for reviews, see [40,41,42,43]). Anticancer activity in various snake venoms has been reported for pancreatic tumor cells [44], human breast cancer (MCF-7), human hepatocellular carcinoma (HepG2), and human prostate carcinoma (DU145) cell lines [45], human leukemic cell lines (U937 and K562), and sarcoma in a Balb C mice model [46], colorectal and breast cancer cell lines [47], human prostate adenocarcinoma (LNCaP), human breast cancer (MCF-7), human colon adenocarcinoma (HT-29), human osteoblastic osteosarcoma (Saos-2) [48], and human breast carcinoma cells (MDA-MB-231 and MCF-7) [49,50].

Literature reports of antitumor effects from venoms derived from invertebrates are less common yet include scorpion venom’s effects on U251-MG glioma cells and xenograft tumors [51], as well as human brain, breast, colorectal, lung, cervix, and larynx cancer cell lines [52]. Bee venom also has reported anticancer effects against MDA-MB-231 and MCF-7 human breast cancer cells [53,54] and ovarian cancers [55,56,57].

Our findings clearly revealed growth-inhibitory activities that were similar among all three SRV extracts examined. The concentration-dependent SRV effects were not markedly different among the three different human cell lines treated (HDFa, SH-SY5Y, and Jurkat E6-1 cells). Interestingly, however, the mouse fibrosarcoma WEHI 164 cell line was remarkably resistant to all three SRVs at the relatively high exposure concentrations and durations tested. Apoptosis was evident by morphological features in HDFa and SH-SY5Y cells treated with eagle ray spine venom (Figure 3). Induction of apoptosis and necrosis by SRV was further confirmed using cell flow cytometry and Jurkat E6-1 cells as targets (Figure 7).

Our live-cell time course analysis further revealed that Atlantic stingray venom evoked both rapid and sustained effects, occurring within minutes after exposure (cell detachment) and persisting for days (growth inhibition and apoptosis). These findings are consistent with the high-content screening results obtained with five different SRV treatments of human cervical cancer HeLa cells [19]. They also align with the antiproliferative effects on HeLa cells previously reported for the crude venom extract from the cowtail stingray (*Pastinachus sephen*, *Dasyatidae* family), which cause enhanced levels of reactive oxygen species, lipid peroxidation, and apoptosis [58]. Both cowtail stingray and spotted eagle ray venoms additionally have reported anticoagulant activity [59].

## 4. Materials and Methods

### 4.1. Capture and Housing of Local Stingrays

Atlantic stingrays (*Hypanus sabinus*, formerly *Dasyatis sabinus*), spotted eagle rays (*Aetobatus narinari*), and cownose rays (*Rhinoptera bonasus*) were captured using small watercraft vessels in nearshore waters off Sarasota, Florida, in Sarasota Bay, or in Terra Ceia Bay, following guidelines specified in the Special Activities Licenses issued to the Mote Marine Laboratory by the Florida Fish and Wildlife Conservation Commission (FFWCC). After visual sighting of stingrays in shallow water, capture was non-invasive, using cast nets for Atlantic stingrays, seine nets for eagle rays, and multi-panel gill nets for cownose rays. Atlantic stingrays and spotted eagle rays were placed in a live well for transport to the laboratory vivarium for later barb collection. Cownose rays were released unharmed after their barbs were collected at time of capture.

Captive Atlantic stingrays were individually housed on a 12 h on/12 h off photoperiod in 340 L holding tanks containing natural seawater maintained at 23–25 °C. Seawater salinity was maintained between 30% and 35% by addition of water that had been de-ionized by reverse osmosis. Periodic exchanges with ozone-treated seawater were performed to supply trace elements and to keep nitrate accumulation below 20 mg/L. Stingrays were fed three times per week with a varied diet of thread herring, shrimp, and squid, supplemented with Elasmo-tabs Vitamins (Mazuri Exotic Animal Diets). Captive eagle rays were housed in a 150,000 L tank containing natural seawater maintained at 25–28 °C and on a 12 h on/12 h off photoperiod. Eagle rays were fed a variety of mollusks daily and were released within 30 days of their capture in compliance with FFWCC regulations.

### 4.2. Spine/Barb Removal Procedures

**Figure 10:**
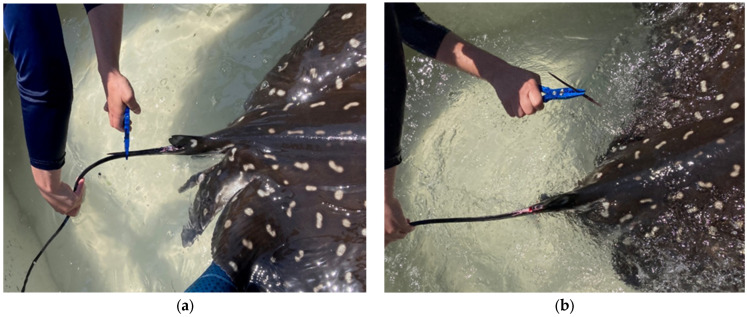
(**a**) Spotted eagle ray spines were removed from the dorsal surface of the tail by first grasping the spine with long nose pliers. (**b**) Twisting the spine dorsal and rostral away from the tail resulted in spine separation from the ray.

Following transport and arrival to the laboratory after capture, spotted eagle ray spines were immediately removed using ethanol-sterilized long nose pliers (Figure 10). Once removed, spines were taken to the laboratory where they were snap frozen and stored at −80 °C. The spotted eagle rays with spines removed were then maintained as described above for approved feeding behavioral studies not associated with this project.

**Figure 11:**
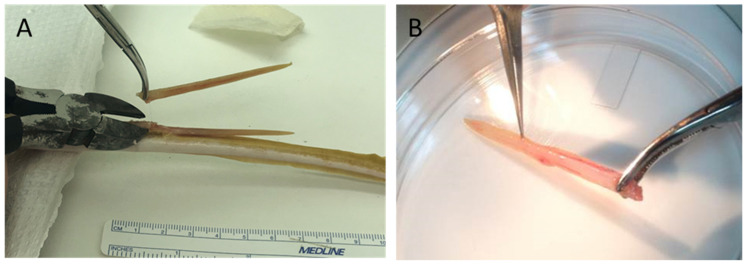
Collection of venom-containing spine tissue: (**A**) Atlantic stingray spines were clipped near their site of attachment on the dorsal surface of the tail using ethanol-sterilized wire-cutting pliers; (**B**) crude venom gland tissue was then scraped from the ventral surface of the spine using sterile fine tip dissecting forceps and into a Petri dish after rinsing with sterile E-PBS solution.

Atlantic stingray spines are more firmly attached to their tail compared to eagle ray spines, and therefore were removed using ethanol-sterilized wire-cutting pliers (Figure 11A). The Atlantic stingrays were anesthetized prior to spine removal to eliminate undue stress on the ray and as a safety precaution for the handler(s). Rays were anesthetized by immersion in seawater containing MS-222 at a concentration of 100–200 mg/L (buffered at 1 part MS-222:2 parts sodium bicarbonate). For some rays, the removed spine was snap frozen and stored at −80 °C for later processing. For others, the freshly removed spine was used immediately for venom tissue collection.

Cownose stingray spines, which are also firmly attached to the tail, were similarly clipped using ethanol-sterilized wire-cutting pliers immediately after their capture. The removed cownose spines were immediately placed in sterile 15 mL tubes and kept on ice during transit to the laboratory where they were then snap frozen and stored at −80 °C for later processing.

As part of their natural turnover cycle, stingray spines are shed (or break off), with new replacement spines growing beneath the functional spine that gets lost. This process continues throughout their lifetime. Clipping stingray spines is analogous to clipping fingernails and toenails on humans and does not injure the animals. Cownose rays and eagle rays were released unharmed after collecting spines. Since these rays were not monitored after release, natural replacement of spines could not be observed.

### 4.3. Collection and Preparation of Crude SRV

The removed spines were initially rinsed with elasmobranch-modified PBS (E-PBS; phosphate-buffered saline modified to an osmolarity of ~970 mOsm by adding NaCl). The venom-containing tissue was then collected by mechanically scraping the ventral side of the barb using sterile forceps (Figure 11B). The collected tissue was then placed in sterile Eppendorf tubes and suspended in 50 mM NH_4_HCO_3_ solution adjusted to pH 7.4. Using a hand-held tissue homogenizer, the tissue was gently homogenized and centrifuged at 10,000× *g* for 10 min. The clear supernatant containing crude SRV extract was then aliquoted into small volumes (100–250 µL). Some aliquots were used for protein quantification and biochemical analysis. Other aliquots were frozen at −80 °C and lyophilized. Lyophilized SRV was stored at −80 °C until assessment for bioactivity.

SDS gels of SRV samples collected from freshly removed spines displayed the same protein pattern as SDS gels of samples collected from spines stored at −80 °C for up to four weeks. Venom samples stored at −80 °C at the time of collection maintained their bioactivity for at least four months, with longer storage times yet to be assessed.

### 4.4. SDS–Polyacrylamide Gel Electrophoresis (PAGE)

Comparative protein profiles of the different SRV extract samples were obtained using SDS-PAGE with 4–20% Mini-PROTEIN TGX Precast gels (Bio-Rad Laboratories, Hercules, CA, USA). Prior to electrophoresis, protein concentrations of the SRV extracts were determined using the Bradford assay (BioRad). SRV samples were loaded at 25 µg protein per lane after denaturation for 5 min at 95 °C. Samples were run at 200 volts and the gels subsequently stained using Coomassie blue for visual analysis.

### 4.5. Cell Culture

To test for SRV bioactivity, four different cell lines of mammalian origin were used as listed in Table 1. All cell lines were obtained from the American Type Culture Collection (ATCC), Manassas, VA, USA. Cells were maintained and sub-cultured according to instructions provided by ATCC. All cell culture media contained antibiotics (penicillin, 100 U/mL/streptomycin, 10 µg/mL). Cell culture reagents were obtained from either Sigma-Aldrich, St. Louis, MO, USA or ATCC.

### 4.6. Cell Growth Assays

The comparative effects of SRVs on cellular growth were determined against all cell types listed in Table 1. Growth-inhibitory activity of cultured cells by the crude SRV extracts was quantified using the MTT assay [15], an assay that measures the ability of live cells to convert the tetrazolium salt MTT (3-(4,5-dimethylthiazol-2-yl)-2,5-diphenyl tetrazolium bromide) to a formazan product via mitochondrial enzymes.

Cells were cultured according to protocols provided by ATCC and adjusted to a concentration of 5 × 10^4^ cells/mL in media with 20% FBS. Cells (100 µL) were added to wells of 96-well microtiter plates in triplicate for control and venom treatments. Venom solutions of varying concentrations in cell culture media were added in 100 µL volumes. For HDFa cells, final well concentrations were 2.5 × 10^4^ cells/mL, 10% FBS, 0–400 µg/mL venom protein. SH-SY5Y cells were used at a concentration of 5 × 10^5^ cells/mL, with final well concentrations of 2.5 × 10^5^ cells/mL, 10% FBS, and 0–200 µg/mL eagle ray venom protein. Cultures were incubated at 37 °C, 5% CO_2_ for 72 h. After the SRV treatment period, a 100 µL supernatant was carefully removed from each well, and 25 µL of MTT solution (5 mg/mL in PBS) was added to each well. For cells in suspension (Jurkat E6-1 cells only), microtiter plates were centrifuged at 300× *g* for 7 min prior to removing the supernatant. Cells were then incubated with MTT for 4 h at 37 °C. After the 4 h incubation period, a solubilizing solution (0.01 N HCl, 10% SDS) was added to each well and incubated overnight at 37 °C.

Using a BioTek Synergy H1 microplate reader, optical density (O.D.) (i.e., absorbance) values were read at 570 nm for each sample with background subtracted at 630 nm. The resulting values for triplicate samples were then averaged. The percent growth inhibition (%GI) was calculated for each treatment as follows: %GI = [1 − (Treatment_Abs570-630_/Control_Abs570-630_)] × 100.

To assess SRV effects on cell morphology, cells from different treatment groups were photographed using an Olympus CK2 inverted microscope and Infinity 2.0 digital camera.

To compare the potency of different SRVs on different cell types, the concentration producing 50% growth inhibition (IC50) was calculated by non-linear least squares regression, where the %GI values at the different concentrations were fit to a Hill function (Equation (1)) using the online Quest Graph™ IC50 Calculator (https://www.aatbio.com/tools/ic50-calculator (accessed on 19 March 2024) [20].

(1) $$Y=Min+\frac{Max-Min}{1+{\left(\frac{X}{IC50}\right)}^{Hill\;coefficient}}$$

### 4.7. Biosensor-Based Real-Time Cell Impedance Recordings

The time course for effects of Atlantic stingray venom was measured on HDFa cells maintained in culture for 5 days using an impedance-based biosensor system (xCELLigence Real-Time Cell Analyzer, ACEA Biosciences) [14]. Time-dependent changes in impedance values reflect changes in cell growth and adherence to the well surface over time [14]. HDFa cells were seeded in 96-well polyethylene terephthalate E-plates (ACEA Biosciences, San Diego, CA, USA) and maintained in culture for up to 5 days at 37 °C, 5% CO_2_. After 3 days in culture, cells were treated with SRV at varying concentrations (0, 1, 3, 10, 30, and 100 μg/mL) in triplicate wells. TritonX-100 at 0.1% was used as a positive control for detaching cells from the well surface. Impedance values for each individual well were recorded every 15 min over the 5-day experimental period. Analysis of the recorded impedance values, quantified as Cell Index (CI) values, was then performed using the system software and data exported to an Excel-compatible file format. The SRV concentration producing a 50% reduction in the normalized CI value (IC50) was also calculated for different exposure periods by non-linear least squares regression using the Hill equation (Equation (1)) and the online Quest Graph™ IC50 Calculator [20].

### 4.8. Flow Cytometry Assay

Cell apoptosis and necrosis were determined using flow cytometry with an annexin V/Dead Cell Apoptosis Kit (Invitrogen, Carlsbad, CA, USA) [24]. Annexin V is a phospholipid-binding protein with a high affinity for phosphatidylserine. In viable healthy cells, annexin V binding is low since phosphatidylserine is located largely on the cytoplasmic surface of cell membranes. In cells undergoing early cell membrane changes associated with apoptosis, phosphatidylserine is translocated from the inner membrane to the outer part of the plasma membrane where it can bind fluorescently labeled annexin V conjugates. Cell death was determined using propidium iodide (PI) that binds DNA accessible in dead and dying cells with compromised cell membranes. Healthy live cells with intact cell membranes prevent PI cellular access and DNA binding.

Jurkat E6-1 cells were plated at 1 × 10^6^ cells per well in a 24-well cell culture plate. SRVs were included with cells in duplicate wells at final protein concentrations of 3.125, 6.25, 12.5, 25, 50, 100, 200, and 400 µg/mL. Staurosporine (2 µM, Sigma) was used as a positive control for apoptosis. Heat-killed cells (95 °C for 30 min) were used as a positive control for necrosis. For each treatment condition, cells were incubated for 24 h at 37 °C, 5% CO_2_.

Following the 24 h treatment period, Jurkat E6-1 cells were transferred to Eppendorf tubes, centrifuged, and washed with ice-cold PBS. Cells were incubated for 15 min with 1 µg/mL PI solution and 5% FITC-labeled annexin V in 0.1 mL annexin binding buffer. Samples were then diluted with 0.4 mL annexin binding buffer and placed on ice. Fluorescence was measured using a flow cytometer (BD Accuri C6 system) at 35 µL/min, where 10,000 events were read with 530 nm excitation and 575 nm emission wavelengths. Unstained controls and cells stained with only PI or FITC were used as instrument controls. Gating criteria for healthy, apoptotic, and necrotic cell populations were set based on positive control values. The percentages of cells counted within each category were then calculated.

### 4.9. Statistical Analyses

Statistical analyses were conducted using SigmaPlot v14.0. Statistical comparisons between experimental groups (e.g., control and SRV-treated cells) were performed using Student’s *t*-test, where *p* values less than 0.05 were considered statistically significant.

## 5. Conclusions

Our comparative analysis of SRV isolated from three local species reveals species-conserved bioactive properties that could have practical biomedical or pharmacological applications. The time course analysis and sensitivity/insensitivity of different cell lines to SRV treatments shed new light with new questions on the cellular processes and underlying molecular mechanisms that mediate their biological activities. Future studies testing individually identified SRV components, e.g., the galectin toxin hypothesis, will fill an important gap in our current knowledge as to which venom-associated proteins recapitulate specific bioactive properties of crude SRV extracts observed in vitro and with envenomation. These ‘next steps’ are essential for future rational design and development of novel therapeutics aimed at better treating the pathophysiological events associated with stingray injuries.

## Figures and Tables

**Table 1:** Cell lines used in bioactivity assays.

Cell Line	ATCC #	Origin	Culture Media	Sub-Culture
HDFa	PCS-201-012	Human, primary dermal fibroblast, normal	Fibroblast Basal Medium (ATCC PCS-201-030); Fibroblast Growth Kit-Low Serum (ATCC PCS-201-041)	At 80–100% confluence
SH-SY5Y	CRL-2266	Human, neuroblastoma	1:1 Eagle’s Minimum Essential Medium/F12 Medium; 10% FBS	4–7 days
Jurkat E6-1	TIB-152	Human, acute T-cell leukemia	RPMI + 10% FBS	2–3 days
WEHI 164	CRL-1751	Mouse, fibrosarcoma	RPMI + 10% FBS	2–3 days

## Data Availability

Data is contained within the article.
